# Reply: Polygenic risk score in cirrhosis: Does the etiology matter?

**DOI:** 10.1097/HC9.0000000000000543

**Published:** 2025-01-16

**Authors:** Tae-Hwi Schwantes-An, Timothy R. Morgan, Devanshi Seth

**Affiliations:** 1 Department of Medical and Molecular Genetics, Indiana University School of Medicine, Indianapolis, Indiana, USA; 2 Department of Medicine, University of California, Irvine, California, USA; 3 Department of Veterans Affairs, VA Long Beach Healthcare System, Long Beach, California, USA; 4 Centenary Institute of Cancer Medicine and Cell Biology, the University of Sydney, Sydney, New South Wales, Australia; 5 Edith Collins Centre (Translational Research in Alcohol Drugs and Toxicology), Sydney Local Health District, Camperdown, New South Wales, Australia; 6 Faculty of Medicine and Health, the University of Sydney, Sydney, New South Wales, Australia

Dear Editor,

We thank Dr Alonso-Peña and colleagues for their comments about genetic risks for alcohol-associated liver cirrhosis (ALC) and for the importance of alcohol intake in the development of cirrhosis among patients with metabolic dysfunction-associated steatotic liver disease (MASLD) with regard to our recent report to *Hepatology Communications*.

We also appreciate that the clinical potential of polygenic score for ALC may extend beyond alcohol-associated liver disease and the importance of an accurate assessment of alcohol consumption in evaluating MASLD, especially in the context of newly minted metabolic and alcohol-associated liver disease (MetALD). We reported that our polygenic score for alcohol-associated liver cirrhosis stratified individuals in the Indiana Biobank Liver cohort for their risk of developing MASLD.^1^ Dr Alonso-Peña and colleagues noted that without an accurate assessment of drinking history in the Indiana Biobank Liver, it would be possible that some of the patients classified as metabolic dysfunction cirrhosis could be either MetALD-cirrhosis or mischaracterized patients with ALC. However, our patients with MASLD cirrhosis were identified by careful clinical chart review led by an expert gastroenterologist and verified as nonalcohol-associated cirrhosis. Also, while occult alcohol use among our patients with MASLD could account for some of the overlap in genetic risks with ALC, we believe that a more likely explanation is that the 2 diseases (MASLD cirrhosis and alcohol-associated cirrhosis) share similar genetic risk factors. Several reviews have highlighted the genetic overlap between MASLD and alcohol-associated liver disease, although we acknowledge that alcohol use was not always assessed in the included studies either.^2^,^3^

Therefore, we too subscribe that assessing alcohol usage among patients with MASLD and categorizing patients into MetALD is important in the context of the true contribution of the shared and independent genetic risks underlying cirrhosis. We are glad Dr Alonso-Peña and colleagues have looked more closely at the important topic that small amounts of alcohol may increase the risk of developing cirrhosis.^4^ Furthermore, it would be nice to have the data to see how well our polygenic score for ALC performs in stratifying risk for developing MetALD to further explore the utility of our score.

Lastly, we agree with Dr Alonso-Peña and colleagues that genetic studies such as ours should be extended to populations beyond those that cluster with 1KGP’s EUR (1000 Genome Project's EUR labeled reference group) ancestry group. Adding diversity in genetic research, especially those that study factors that are influenced by social determinants of health, such as alcohol consumption, would further improve the generalizability and identify population-specific and cross-population effectors of disease risk.

Again, we thank Dr Alonso-Peña and colleagues for emphasizing the importance of assessing alcohol consumption in MASLD studies and look forward to their report. We hope to have opportunities in the near future to address these critical issues in our field via the availability of a more diverse data set with appropriate assessment data.
